# The Hamming Ball Sampler

**DOI:** 10.1080/01621459.2016.1222288

**Published:** 2017-09-03

**Authors:** Michalis K. Titsias, Christopher Yau

**Affiliations:** ^a^ Department of Informatics, Athens University of Economics and Business, Athens, Greece; ^b^ Wellcome Trust Centre for Human Genetics, University of Oxford, Oxford, United Kingdom; ^c^ Department of Statistics, University of Oxford, Oxford, United Kingdom

**Keywords:** Bayesian, Discrete state spaces, Markov chain Monte Carlo

## Abstract

We introduce the Hamming ball sampler, a novel Markov chain Monte Carlo algorithm, for efficient inference in statistical models involving high-dimensional discrete state spaces. The sampling scheme uses an auxiliary variable construction that adaptively truncates the model space allowing iterative exploration of the full model space. The approach generalizes conventional Gibbs sampling schemes for discrete spaces and provides an intuitive means for user-controlled balance between statistical efficiency and computational tractability. We illustrate the generic utility of our sampling algorithm through application to a range of statistical models. Supplementary materials for this article are available online.

## 1. Introduction

Statistical inference of high-dimensional discrete-valued vectors or matrices underpins many problems across a variety of applications including language modeling, genetics, and image analysis. Bayesian approaches for such models typically rely on the use of Markov chain Monte Carlo (MCMC) algorithms to simulate from the posterior distribution over these objects. The effective use of such techniques requires the specification of a suitable proposal distribution that allows the MCMC algorithm to fully explore the discrete state space while maintaining sampling efficiency. While there have been intense efforts to design optimal proposal distributions for continuous state spaces, generic approaches for high-dimensional discrete state models have received relatively less attention but some examples include the classic Swendsen–Wang algorithm (Swendsen and Wang 1987) for Ising/Potts models and more recent sequential Monte Carlo methods (Schäfer and Chopin 2013).

In this article, we consider Bayesian inference using MCMC for an unobserved latent discrete-valued discrete sequence or matrix X∈X, where each element *x_ij_* ∈ {1, …, *S*}, given observations **y** = [*y*_1_, …, *y_N_*]. We will assume that the observations are conditionally independent given **X** and model parameters θ so that the joint distribution factorizes as *p*(**y**, **X**, θ) = [∏^*N*^_*i* = 1_ *p*(*y_i_*|**X**, θ)]*p*(**X**, θ). We further assume that the posterior distribution *p*(**X**, θ|**y**) has a complex dependence structure so that standard MCMC schemes, such as a (Metropolis-within) Gibbs Sampler, using

(1) θ←p(θ|X,y),[3pt]

(2) X←p(X|θ,y),

or a marginal Metropolis–Hastings sampler over θ based on

(3) $$\theta \leftarrow p\left(\theta |y\right)\propto \sum_{X\in X}p\left(y,X,\theta \right),$$

are both intractable because exhaustive summation over the entire state space of **X** has exponential complexity.

A popular and tractable alternative is to employ block-conditional (Metropolis-within) Gibbs sampling in which subsets **x**_*i*_ of **X** are updated conditional on other elements being fixed using

(4) θ←p(θ|X,y),

(5) $$x_{i}\leftarrow p\left(x_{i}|X_{-i},\theta ,y\right),\forall i,$$

where **X**_− *i*_ denotes the elements excluding those in **x**_*i*_. Typical block structures might be rows/columns of **X**, when it is a matrix, or sub-blocks when **X** is a vector. While block-conditional sampling approaches are often convenient (they may be of closed form allowing for Gibbs sampling without resort to Metropolis–Hastings steps), in high dimensions, major alterations to the configuration of **X** maybe difficult to achieve as this must be done via a succession of small (possibly low probability) incremental changes. Conditional sampling may lead to an inability to escape from local modes in the posterior distribution particularly if the elements of **X** exhibit strong correlations with each other and together with θ.

To address these problems, we propose a novel and generic MCMC sampling procedure for high-dimensional discrete-state models, named the “Hamming ball sampler.” This sampling algorithm employs auxiliary variables that allow iterative sampling from slices of the model space. Marginalization within these model slices is computationally feasible and, by using sufficiently large slices, it is also possible to make significant changes to the configuration of **X**. The proposed sampling algorithm spans a spectrum of procedures that contains the marginal and block-conditional Gibbs sampling strategies as extremes. At the same time, it allows the user to express many more novel schemes so that to select the one that best balances statistical efficiency and computational tractability.

We demonstrate the utility of the sampling procedure with three different statistical models where exhaustive enumeration is impossible for realistic datasets and illustrate the considerable benefits over standard sampling approaches.

## 2. Theory

In this section, we describe the theoretical foundations of the proposed sampling algorithm (Sections 2.1– 2.4) and we discuss computational complexity and sampling efficiency (Section 2.5).

### 2.1 Construction

The Hamming ball sampler considers an augmented joint probability model that can be factorized as *p*(**y**, **X**, θ, **U**) = *p*(**y**, **X**, θ)*p*(**U**|**X**), where the extra factor *p*(**U**|**X**) is a conditional distribution over an auxiliary variable **U**, which lives in the same space and has the same dimensions as **X**. The conditional distribution *p*(**U**|**X**) is chosen to be a uniform distribution over a neighborhood set $H_{m}\left(X\right)$ centered at **X**,

(6) $$p\left(U|X\right)=\frac{1}{Z_{m}}I\left(U\in H_{m}\left(X\right)\right),$$

where I(·) denotes the indicator function and the normalizing constant *Z_m_* is the cardinality of $H_{m}\left(X\right)$.

The neighborhood set $H_{m}\left(X\right)$ will be referred to as a *Hamming ball* since it is defined through Hamming distances so that

(7) $$H_{m}\left(X\right)=\left\{U:d\left(u_{i},x_{i}\right)\leq m,i=1,\ldots ,P\right\}.$$

Here, *d*(**x**_*i*_, **u**_*i*_) denotes the Hamming distance $\sum_{j}I\left(u_{ij}\neq x_{ij}\right)$ and the pairs (**u**_*i*_, **x**_*i*_) denote nonoverlapping subsets of corresponding entries in (**U**, **X**) such that ∪^*P*^_*i* = 1_ **u**_*i*_ = **U** and ∪^*P*^_*i* = 1_ **x**_*i*_ = **X**. Also, the parameter *m* denotes the maximal distance or radius of each individual Hamming ball set. For instance, these pairs can correspond to different matrix columns so that **x**_*i*_ will be the *i*th column of **X** and **u**_*i*_ the corresponding column of **U**. Hence, the Hamming ball $H_{m}\left(X\right)$ would consist of all matrices whose columns are *at most* *m* elements different to **X**.

Furthermore, the auxiliary factor *p*(**U**|**X**) factorizes across the *P* blocks {**u**_*i*_, **x**_*i*_}^*P*^_*p* = 1_ as follows:

(8) $$p\left(U|X\right)=\prod_{i=1}^{P}p\left(u_{i}|x_{i}\right)=\prod_{i=1}^{P}\frac{1}{Z_{i,m}}I\left(\text{d}\left(u_{i},x_{i}\right)\leq m\right),$$

where *Z*_*i*, *m*_ is the volume of the individual Hamming ball set $H_{m}\left(x_{i}\right)=\left\{u_{i}:d\left(u_{i},x_{i}\right)\leq m\right\}$, which, in case each block **x**_*i*_ consists of *K* elements, is equal to Zi,m=M=∑j=0m(S-1)jKj. Importantly, the *Z*_*i*, *m*_ is *independent* of the exact values of **u**_*i*_ and **x**_*i*_ (i.e., the volume of the Hamming ball does not depend on where we are in the model space). This is critical for the application of Gibbs sampling later on.

Notice that the above factorization is just a consequence of the product factorization of the indicator function $I(U\in H_{m}\left(X\right))$ across the blocks. More precisely, this indicator function satisfies the following factorization and symmetry properties:

(9) $$I\left(U\in H_{m}\left(X\right)\right)=\prod_{i=1}^{P}I\left(\text{d}\left(u_{i},x_{i}\right)\leq m\right),$$

(10) $$I\left(U\in H_{m}\left(X\right)\right)=I\left(X\in H_{m}\left(U\right)\right),\;\forall X,U\in X,$$

where the second one is direct consequence of the symmetry of the Hamming distance $\text{d}(u_{i},x_{i})$.

### 2.2 Gibbs Sampling

The principle behind the Hamming ball sampler is that the use of Gibbs sampling for the augmented joint probability distribution *p*(**y**, **X**, θ, **U**) admits the *target* posterior distribution *p*(**X**, θ|**y**) as a by-product (since marginalization over **U** recovers the target distribution). Specifically, the Hamming ball sampler alternates between the steps:

(11) U←p(U|X),[4pt]

(12) (θ,X)←p(θ,X|y,U).

The update of (θ, **X**) can be implemented as two conditional (Gibbs) updates:

(13) θ←p(θ|X,y),[4pt]

(14) X←p(X|θ,U,y).

Or, alternatively, as a joint update via a Metropolis–Hastings accept–reject step that draws a new (θ′, **X**′) from the proposal distribution *Q*(θ′, **X**′|θ, **X**) = *p*(**X**′|θ′, **U**, **y**)*q*(θ′|θ) and accepts it with probability

(15) $$\begin{array}{ccc} & & \min\left(1,\frac{p(y,X^{'},\theta^{'},U)}{p(y,X,\theta ,U)}\frac{p\left(X|\theta ,U,y\right)q\left(\theta |\theta^{'}\right)}{p\left(X^{'}|\theta^{'},U,y\right)q\left(\theta^{'}|\theta \right)}\right)\\ & & \;=\min\left(1,\frac{p(\theta^{'},U,y)}{p(\theta ,U,y)}\frac{q(\theta |\theta^{'})}{q(\theta^{'}|\theta )}\right), \end{array}$$

where *q*(θ′|θ) is a proposal distribution over the model parameters.

### 2.3 Restricted State Space

Crucially, the restricted state space defined by the Hamming ball, which has been injected into the model via the auxiliary factor *p*(**U**|**X**), means that the conditional distribution *p*(**X**|θ, **U**, **y**) can be[Fig f0001]tractably computed as

(16) $$p\left(X|\theta ,U,y\right)=\frac{p(y,X,\theta )p(U|X)}{p(\theta ,U,y)}=\frac{p\left(y,X,\theta \right)I(X\in H_{m}\left(U\right))}{\tilde{p}\left(\theta ,U,y\right)},$$

where we used Equation (10), $p\left(\theta ,U,y\right)=\frac{\tilde{p}\left(\theta ,U,y\right)}{Z_{m}}$ and $\tilde{p}\left(\theta ,U,y\right)=\sum_{X^{'}\in H_{m}\left(U\right)}p\left(y,X^{'},\theta \right)$ is the normalizing constant found by exhaustive summation over all admissible matrices inside the Hamming ball $H_{m}\left(U\right)$. Through careful selection of *m*, the cardinality of $H_{m}\left(U\right)$ will be considerably less than the cardinality of X so that exhaustive enumeration of all elements inside the Hamming ball would be computationally feasible.

**Figure 1. f0001:**
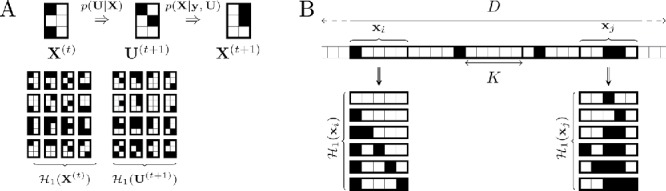
Hamming ball sampler illustration. Panel (a) illustrates a Hamming ball update (*m* = 1) for a 2 × 3 binary matrix **X** ^(*t*)^ to **X** ^(*t* + 1)^ via **U** ^(*t* + 1)^ where the subsets (**x**, **u**) correspond to columns of the matrix. Panel (b) illustrates a block strategy for the application of Hamming ball sampling when **X** is a *D* × 1 vector split into random blocks of size *K*.

Overall, the proposed construction uses the auxiliary variable **U** to define a *slice* of the model given by $H_{m}\left(U\right)$. Sampling of (θ, **X**) is performed within this sliced part of the model through *p*(θ, **X**|**y**, **U**). At each iteration, this model slice randomly moves via the resampling of **U** in step (11), which simply sets **U** to a random element from $H_{m}\left(X\right)$. This resampling step allows for *random exploration* that is necessary to ensure that the overall sampling scheme is ergodic. The amount of exploration depends on the radius *m* so that **U** can differ from the current state of the chain, say **X** ^(*t*)^, at most in *mP* elements, that is, the maximum Hamming distance between **U** and **X** ^(*t*)^ is *mP*. Similarly, the subsequent step of drawing the new state, say **X** ^(*t* + 1)^, is such that at maximum **X** ^(*t* + 1)^ can differ from **U** in *mP* elements and overall it can differ from the previous **X** ^(*t*)^ at most in 2*mP* elements. Figure 1(a) graphically illustrates the workings of the Hamming ball sampler.

Furthermore, it is worth noting that when the restriction in the state space is relaxed, that is, when the radius *m* becomes large enough, the Hamming ball samplers reduce to the (Metropolis-within) Gibbs and marginal schemes outlined in (1)–(2) and (3). More precisely, if the size of each block **x**_*i*_ is *K* and we assume a Hamming radius equal to the block size, that is, *m* = *K*, then the Hamming ball set $H_{m}\left(U\right)$ is completely unrestricted and becomes equal to X. In such case the restricted conditional *p*(**X**|θ, **U**, **y**) becomes equal to the exact (unrestricted) conditional *p*(**X**|θ, **y**) since $I(X\in H_{m}\left(U\right))=1$ for any X,U∈X. Similarly, the restricted $\tilde{p}\left(\theta ,U,y\right)$ normalizing constant of *p*(**X**|θ, **U**, **y**) reduces to the exact marginal *p*(θ, **y**) and therefore the Hamming ball sampler schemes reduce to the algorithms described by (1)–(2) and (3), respectively.

### 2.4 Selection of Blocks

The application of the Hamming ball sampler requires the selection of the subsets or blocks {**x**_1_, …, **x**_*P*_}. This selection will depend on the conditional dependencies specified by the statistical model underlying the problem to be addressed. For some problems, such as the tumor deconvolution mixture model considered later, there may exist a natural choice for these subsets (e.g., columns of a matrix) that can lead to efficient implementations. For instance, under a suitable selection of blocks the posterior conditional *p*(**X**|θ, **U**, **y**) could be fully factorized, that is, *p*(**X**|θ, **U**, **y**) = ∏^*P*^_*i* = 1_ *p*(**x**_*i*_|θ, **u**_*i*_, **y**), or have a simple Markov dependence structure so that exact simulation of **X** would be feasible. In contrast, for unstructured models, where **X** is just a large pool of fully dependent discrete variables (stored as a *D*-dimensional vector), we can divide the variables into randomly chosen blocks **x**_*i*_, *i* = 1, …, *P*, so that they have equal length $K=\text{length}(x_{i})$. (If *D*/*P* is not an integer, then the final block **x**_*P*_ will have size smaller than *K*.) In such cases, exact simulation from *p*(**X**|θ, **U**, **y**) may not be feasible and instead we can use the Hamming ball operation to sequentially sample each block. More precisely, this variant of the algorithm can be based on the iteration (11), (13)–(14) with the only difference that the steps (11) and (14) are now split into *P* sequential conditional steps,

(17) $$u_{i}\leftarrow p\left(u_{i}|x_{i}\right),\;x_{i}\leftarrow p\left(x_{i}|X_{-i},\theta ,u_{i},y\right),\forall i,$$

where the posterior conditional *p*(**x**_*i*_|**X**_− *i*_, θ, **u**_*i*_, **y**) simplifies to

(18) $$p\left(x_{i}|X_{-i},\theta ,u_{i},y\right)\propto p\left(y,x_{i},X_{-i},\theta \right)I\left(\text{d}\left(u_{i},x_{i}\right)\leq m\right),$$

where we made use of the factorization of the auxiliary distribution *p*(**U**|**X**) from (8). The above scheme can be thought of as a *block Hamming ball sampler*, which incorporates standard block Gibbs sampling (see iterations (4)–(5)) as a special case obtained when the radius *m* is equal to the block size *K*. In a purely block Hamming ball scheme we will have *m* < *K* and in general the parameters (*m*, *K*) can be used to jointly control algorithmic performance (illustrative examples are given in Section 3.1 and in the supplementary information at Section S1). This scheme is illustrated in Figure 1(b) and used later in the sparse linear regression application.

### 2.5 Computational Time Complexity and Sampling Efficiency

We now discuss the relative computational time complexity of the Hamming ball sampler compared to the block Gibbs sampler. For simplicity, we assume that the conditional posterior distribution *p*(**X**|θ, **U**, **y**) factorizes across *P* blocks of size *K*. The computational time complexity of the Hamming ball sampler scales with the Hamming radius *m*, block size *K*, and the number of blocks *P* according to *O*(*MP*) where M=∑j=0m(S-1)jKj. The standard block Gibbs sampler is a special case of the Hamming ball sampler where the Hamming distance is always the same as the block size (*m* = *K*) and therefore has computational time complexity of *O*(*S^K^P*) (where SK=∑j=0K(S-1)jKj). The block Gibbs sampler is therefore only practical for small values of block size *K* since we must enumerate all possible values for that block in each Gibbs sampling sweep. The Hamming ball sampler provides flexibility in that we can choose to use larger block sizes *K* and simultaneously use a smaller Hamming distance *m* to limit the possible values to maintain a manageable computational time cost. However, to understand if this flexibility can prove useful, we must also consider the relative sampling efficiencies of the two approaches.

Sampling efficiency characterizes the sampler in terms of its ability to fully explore the probability distribution and traverse between different modes. It depends on both the algorithm as well as the landscape of the probability distribution under exploration. Therefore, an ideal sampling algorithm needs to strike a balance between computational time complexity and sampling efficiency to realize the largest number of (effective) independent posterior samples in each unit of computational time. We use the following measure to quantify overall efficiency:

$$\begin{array}{c} \text{Overall}\;\text{efficiency}\propto \frac{\#\text{Effective}\;\text{sample}\;\text{size}\;\text{in}\;n\;\text{iterations}}{\text{time}\;\text{to}\;\text{perform}\;n\;\text{iterations}}=\frac{s_{n}}{T_{n}}, \end{array}$$

which expresses a balance between how effective the algorithm is in producing independent samples and how fast it runs. Notice that for real inference problems it is ordinarily very difficult to compute the above measure (in particular, when the posterior distribution has an unknown number of modes, the computation of the effective sample size is very challenging and standard autocorrelation measures can be misleading) to rank the different sampling schemes. For benchmarking purposes, we can develop simulated scenarios where the properties of the posterior landscape can be determined in advanced. Using an illustrative example, similar to that given in Section 3.1, in which the true posterior distribution was bimodal, we applied a range of Hamming ball sampling schemes with different combinations of parameters (*m*, *K*) to explore the posterior. This included schemes where *m* = *K* that correspond to block Gibbs samplers. We approximated *s_n_* by counting the number of times each sampling scheme was able to switch from one mode to the other.

Supplementary Figure S1 shows the performance for each of the sampling schemes and we note that the overall best performing sampler was for (*m*, *K*) = (1, 13) and none of the block Gibbs samplers were among the most effective sampling schemes. We concluded that while block Gibbs samplers, for a given block size, had the best sampling efficiency, the need to consider *all* enumerations of the latent variables means they also have the worse computational time complexity. We shall discuss in further detail in the forthcoming examples but, by limiting the number of computations with the Hamming distance criterion, the Hamming ball samplers can achieve a better balance between the computational complexity and sampling efficiency.

## 3. Examples

We now illustrate the utility of our Hamming ball sampling scheme through its application to two statistical models: in mixture modeling for tumor deconvolution (Section 3.3) and Bayesian variable selection in linear regression (Section 3.2). We also give an example based on factorial hidden Markov models in the supplementary information. Simulated examples also are used to characterize the properties of the Markov chain Monte Carlo sampling approaches tested. Our emphasis is on evaluating the performance of Hamming ball-based sampling versus conventional block Gibbs sampling approaches rather than the quality of the model specifications. However, we first illustrate the use of the Hamming ball sampler through a simulated toy example.

### 3.1 An Illustrative Toy Example

Here, we give a simple example that illustrates the differences between the Hamming ball sampler and a standard Gibbs sampler.

We consider a simple observation model that generates a scalar output *y_i_* given a vector of covariates **z**_*i*_ according to

(19) $$y_{i}=\sum_{d=1}^{N}x_{d}z_{i,d}+\eta_{i},\;\eta_{i}∼N\left(0,\sigma^{2}\right),$$

where **X** = (*x*_1_, …, *x_D_*) is a latent binary vector that follows a prior distribution such that $\text{Pr}(x_{d}=1)=0.5$, *d* = 1, …, *D*. This is a simplified version of a variable selection problem in linear regression where, for instance, the regression coefficients are assumed to have known values that are fixed to unity. Given a set of examples {*y_i_*, **z**_*i*_}^*n*^_*i* = 1_, our goal is to estimate the posterior distribution over **X** while the parameter σ^2^ is also taken as known. In the next section, we will consider a much more realistic variable selection problem, but the current toy model suffices to clearly illustrate the differences between the Hamming ball sampler and the standard Gibbs sampler.

For this illustration, we simulated datasets where *n* = 200, *D* = 20 and the covariates for the *i*th example where such that *z*_*i*, *d*_ ∼ *U*(0, 1) with *d* = 1, …, 10 while the remaining covariates where exact replicas, that is, *z*_*i*, 10 + *d*_ = *z_d_*, *d* = 1, …, 10. Then, each response was generated according to $y_{i}=z_{i,6}+\eta_{i},\;\eta ∼N\left(0,\sigma^{2}\right)$. The overall simulation creates two completely symmetric modes in the posterior distribution over **X** so that the 6th and the 16th covariates could provide equally good explanation of the observed responses. More precisely, the first mode is such that (*x*_6_ = 1, *x*_16_ = 0) and the second one is such that (*x*_6_ = 0, *x*_16_ = 1), while for both modes the remaining *x_d_*’s are zero.

We simulated three different datasets by varying the level of the noise variance according to σ^2^ = 0.5, 2, 5. In the first case (σ^2^ = 0.5), we have a very informative likelihood function that induces a posterior distribution over **X** where the two modes are very sharply picked and there is little probability in the regions between these modes. In the second case (σ^2^ = 2), we have a more diffuse posterior distribution where the two modes are less sharply picked, while in the third case (σ^2^ = 5) the posterior becomes even more diffuse and closer to the uniform prior.

**Figure 2.**
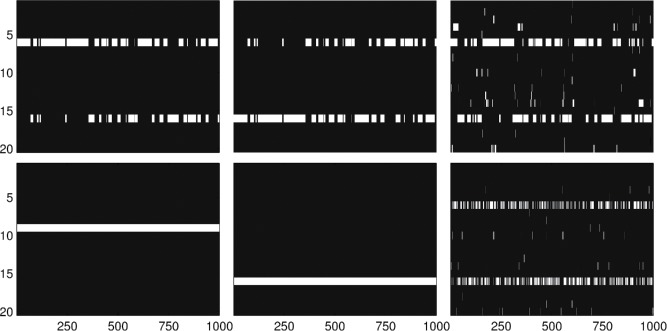
Illustrative toy example simulation. The evolution of a 20-dimensional binary state vector (see Section 3.1), where zero values are shown in black and ones in white, for 1000 sampling iterations obtained by the (top row) Hamming ball sampling and (bottom row) Gibbs sampling. Variables 6 or 16 are relevant to the regression and the sampler should switch between the inclusion of one or the two. The corresponding two plots in each column (from left to right) correspond to the three different values of the noise variance: σ^2^ = 0.5, 2, 5.

To estimate the posterior over **X**, we consider the simplest possible Hamming ball sampler that jointly samples **X** using a Hamming ball of radius *m* = 1, and also the simplest possible Gibbs sampler that in each iteration sequentially samples an *x_d_* conditional on the remaining elements of **X**. Both samplers have the same computational complexity *O*(*D*) and the actual running times are roughly the same. They essentially differ on how they allocate their computational resources. Figure 2 shows the evolution of the state vector **X** for both algorithms and all three cases when performing 1000 sampling iterations (collected after 100 burn-in samples). Clearly, the Hamming ball sampler is able to mix well between the modes in all three cases, since it is able to simultaneously make at most 2*m* = 2 changes in **X** during each sweep, and provides a correct estimation of the posterior distribution.

In contrast, the Gibbs sampler becomes stuck in local posterior modes in the first two instances as it cannot simultaneously flip two bits in **X** and jump between modes. The Gibbs sampler is only able to mix well in the presence of large observation noise variance σ^2^ = 5 when the posterior distribution is diffuse and there is sufficient probability mass for the Gibbs sampler to traverse between the two posterior modes. In this case, the posterior distribution is near-independent in the latent variables and the Gibbs sampler becomes very efficient since it performs exhaustive local exploration by giving a chance to all *D* bits to get flipped in a full sweep. The Hamming ball sampler can flip less than *D* bits in a single sweep, and therefore for very diffuse posterior distributions it will be less effective than Gibbs sampling. In contrast, for correlated posterior distributions Hamming ball sampling can be more effective than Gibbs sampling since it has the ability to perform joint updates and jump between modes.

The above simple illustrative example demonstrates the potential advantages of the Hamming ball sampler over standard Gibbs approaches. In practice, a mixture of Gibbs and Hamming ball sampling moves could be used to balance global and local exploration of the posterior distribution.

### 3.2 Sparse Linear Regression

We now consider variable selection problems with sparse linear regression models. Applications of such models can arise in problems such as expression quantitative trait loci (eQTL) analysis that is concerned with the association of genotype measurements, typically single nucleotide polymorphisms (SNPs), with phenotype observations (see, e.g., O’Hara et al. 2009; Bottolo and Richardson 2010; Peltola, Marttinen, and Vehtari 2012, and references therein). The interest lies in the posterior distribution over some binary vector **X** that encodes the selection over the covariates and can inform us as to which covariates are most important for defining the observed response variable. Typically **X** is assumed to be sparse so that only a few covariates contribute to the explanation of the observations.

#### 3.2.1 Setup

We consider a dataset {*y_i_*, **z**_*i*_}^*N*^_*i* = 1_ where $y_{i}\in R$ is the observed response and $z_{i}\in R^{D}$ is the vector of the corresponding covariates. We can collectively store all responses in an *N* × 1 vector **y** (assumed to be normalized to have zero mean) and the covariates in an *N* × *D* design matrix **Z**. We further assume that from the total *D* covariates there exists a small unknown subset of relevant covariates that generates the response. This is represented by a *D*-dimensional unobserved binary vector **X** that indicates the relevant covariates and follows an independent Bernoulli prior distribution,

$$\begin{array}{c} x_{d}∼\mathrm{Bernoulli}\left(x_{d},\pi_{0}\right),\;\;d=1,\ldots ,D, \end{array}$$

where π_0_ is assigned a conjugate Beta prior, $\text{Beta}(\pi_{0}|\alpha_{\pi_{0}},b_{\pi_{0}})$, and $\left(\alpha_{\pi_{0}},b_{\pi_{0}}\right)$ are hyperparameters. Given **X**, a Gaussian linear regression model takes the form

$$\begin{array}{c} y=Z_{X}\beta_{X}+\eta ,\;\;\eta ∼N\left(0,\sigma^{2}I_{N}\right), \end{array}$$

where **Z**_**X**_ is the *N* × *D*_**X**_ design matrix, with *D*_**X**_ = ∑^*D*^_*d* = 1_ *x_d_*, having columns corresponding to *x_d_* = 1 and $\beta_{X}$ is the respective *D*_**X**_ × 1 vector of regression coefficients. The regression coefficients $\beta_{X}$ and the noise variance σ^2^ are assigned a conjugate normal-inverse-gamma prior

$$\begin{array}{c} p(\beta_{X},\sigma^{2}|X)=N(\beta_{X}\left|0,g(Z_{X}^{T}Z_{X}{)}^{-1})\text{InvGa}(\sigma^{2}\right|\alpha_{\sigma },b_{\sigma }), \end{array}$$

where (*g*, α_σ_, *b*_σ_) are hyperparameters. Notice that the particular choice *g*(**Z** ^*T*^_**X**_ **Z**_**X**_)^− 1^ for the covariance matrix, where is *g* is scalar hyperparameter, corresponds to the so-called *g*-prior (Zellner 1986).

#### 3.2.2 Posterior Inference

Based on the above form of the prior distributions we can analytically marginalize out the parameters $\theta =(\pi_{0},\beta_{x},\sigma^{2})$ and obtain the marginalized joint density (Bottolo and Richardson 2010):

$$\begin{array}{c} p\left(y,X|\cdot \right)\propto C{\left(2b_{\sigma }+S\left(X\right)\right)}^{-(2\alpha_{\sigma }+N-1)/2}, \end{array}$$

where

$$\begin{array}{ccc} C & = & {\left(1+g\right)}^{-D_{X}/2}\Gamma \left(D_{X}+\alpha_{\pi_{0}}\right)\Gamma \left(D-D_{X}+b_{\pi_{0}}\right),\\ S(X) & = & y^{T}y-\frac{g}{1+g}y^{T}Z_{X}(Z_{X}^{T}Z_{X}{)}^{-1}Z_{X}^{T}y, \end{array}$$

and Γ( · ) denotes the Gamma function. The hyperparameters of the prior were set to fixed values as follows. The hyperparameters of $\text{InvGa}(\sigma^{2}|\alpha_{\sigma },b_{\sigma })$ were set to α_σ_ = 0.1 and *b*_σ_ = 0.1, which leads to a vague prior. The scalar hyperparameter for the *g*-prior were chosen to *g* = *N* as also used by Bottolo and Richardson (2010). Finally, the hyperparameters for the Beta prior, $\text{Beta}(\pi_{0}|\alpha_{\pi_{0}},b_{\pi_{0}})$, were set to the values $\alpha_{\pi_{0}}=0.001$ and $b_{\pi_{0}}=1$, which favors sparse configurations for the vector **X**.

The sampling algorithm we use is a block Hamming ball sampler, which consists of a combination of a block Gibbs sampler with Hamming ball moves. More precisely, at each iteration where the current value of **X** is **X** ^(*t*)^, we randomly divide the *D* covariates into blocks of size *K* so that we have *P* = *D*/*K* separate blocks. Then, we iteratively visit each block **x**_*i*_ and sample a new value for its elements based on a Hamming ball move. The whole scheme follows the iteration given below:

1. Randomly initialize **X** ^(0)^ and set *t* = 0.
2. At iteration *t* + 1 = 1, …, *T* randomly divide the elements in **X** into *P* = *D*/*K* random blocks so that **x**_*i*_ denotes the elements of the *i*th block.
3. for *i* = 1, …, *P*
  (a) Sample auxiliary variables **u** ^(*t* + 1)^_*i*_:
    (20) $$\begin{array}{ccc} & & \;\;\;\;\;\;\;\;u_{i}^{\left(t+1\right)}∼p\left(u_{i}^{\left(t+1\right)}|x_{i}^{\left(t\right)}\right)\\ & & \;\;\;\;\;\;\;\;\;=\frac{1}{\sum_{u_{i}^{\left(t+1\right)}\in H_{m}\left(x_{i}^{\left(t\right)}\right)}1},\forall u_{i}^{\left(t+1\right)}\in H_{m}\left(x_{i}^{\left(t\right)}\right). \end{array}$$
  (b) Sample **x** ^(*t* + 1)^_*i*_:
    (21) $$\begin{array}{ccc} & & \;\;\;\;\;\;\;\;\;\;\;\;\;\;\;\;\;\;\;\;\;\;\;\;\;\;\;\;\;\;\;\;\;\;\;\;\;\;\;\;\;\;\;\;\;\;\;\;x_{i}^{\left(t+1\right)}∼\\ & & \;\;\;\;\;\;\;\;\;\;\;\;\;\;\;\;\;\;\;\;\;\;\;\;\;\;\;\;\;\;\;\;\;\;\;\;\;\;\;\;\;\;\;\;\;\;\;\;\;\;\frac{p(y,x_{1}^{\left(t+1\right)},\ldots ,x_{i}^{\left(t+1\right)},x_{i+1}^{\left(t\right)},\ldots ,x_{P}^{\left(t\right)}|g,\alpha_{\sigma },b_{\sigma },b_{\pi_{0}},\alpha_{\pi_{0}})}{\sum_{x_{i}^{\left(t+1\right)}\in H_{m}\left(u_{i}^{\left(t+1\right)}\right)}p\left(y,x_{1}^{\left(t+1\right)},\ldots ,x_{i}^{\left(t+1\right)},x_{i+1}^{\left(t\right)},\ldots ,x_{P}^{\left(t\right)}|g,\alpha_{\sigma },b_{\sigma },b_{\pi_{0}},\alpha_{\pi_{0}}\right).} \end{array}$$

The above block Hamming ball sampler was applied to all examples assuming a fixed block size *K* = 10 and different values for the Hamming radius, *m* = 1 − 3 (named HB1-3). We also applied various block Gibbs samplers, which sample between 1 and 3 elements (named BG1-3) of **X** at a time and are obtained as special cases of the block Hamming ball sampling algorithm by setting *K* = *m*. For all MCMC algorithms, we used a burn-in phase of 100 iterations and then we collected 100,000 samples during the main sampling phase.

#### 3.2.3 Results

We simulated a regression dataset with *N* = 100 responses and *D* = 1200 covariates in which there were two relevant covariates that fully explain the data while the remainder were noisy redundant inputs. As a consequence this sets up a challenging model exploration problem as only two out of 2^1200^ possible models represent the possible truth.

We did this by first generating a 100 × 600 design matrix **Z** such that [**Z**]_*ij*_ is sampled uniformly from {0, 1, 2}. We set the binary latent vector **X** to be everywhere zero apart from *x*_11_ = 1, which was the only relevant covariate. The regression coefficients β were set to the vector of ones and the responses were generated according to

(22) $$y=Z_{X}\beta_{X}+\eta ,\;\;\eta ∼N\left(0,0.1^{2}I_{N}\right),$$

or

(23) $$y_{i}=z_{i,11}+\eta ,\;\;\eta_{i}∼N\left(0,0.1^{2}\right),\;\;i=1,\ldots ,N.$$

Subsequently, to create a completely symmetric mode in the posterior distribution over **X**, we replicated the covariates so that to finally obtain a new design matrix **Z** = [**Z**, **Z**], which had size 100 × 1200. In this way the 11th and the 611th covariates could provide equally good explanations of the observed responses **y** and therefore an efficient MCMC algorithm should identify both covariates and switch them frequently during sampling.

**Figure 3.**
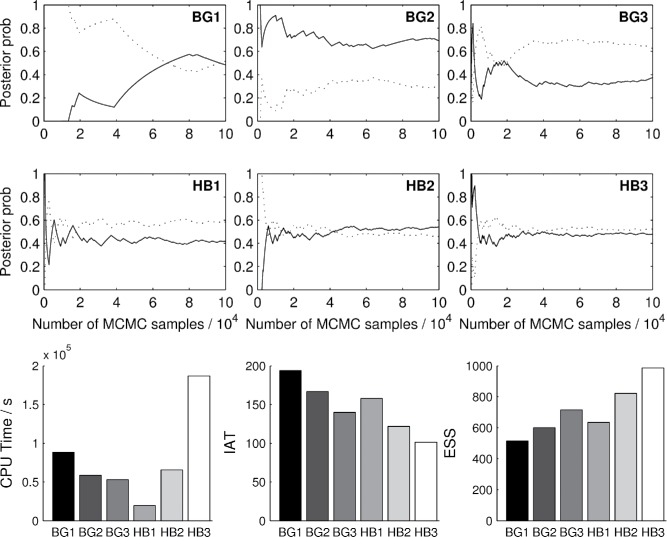
Comparison of block Gibbs and Hamming ball sampling schemes for the simulation regression example. Top and middle rows give trace plots showing the running marginal posterior inclusion probabilities for **x**_11_ (solid) and **x**_611_ (dashed). Bottom row shows CPU times, integrated autocorrelation times (IAT), and effective sample size (ESS) estimates for each method.

We applied our MCMC sampling schemes to sample from the posterior distribution over this massive space of possible models. Figure 3 [Fig f0004]compares the relative performance of the various sampling schemes. The trace plots show the running marginal posterior inclusion probabilities of the two relevant variables *x*_11_ and *x*_611_, which converge to the expected values of 0.5 with the Hamming ball samplers but not with the block Gibbs samplers. This indicates that the Hamming ball schemes were mixing well, able to identify the two relevant variables and frequently switched between their inclusions. In contrast, the block Gibbs samplers exhibited strong correlation effects (stickiness) that impaired their efficiency.

**Figure 4. f0004:**
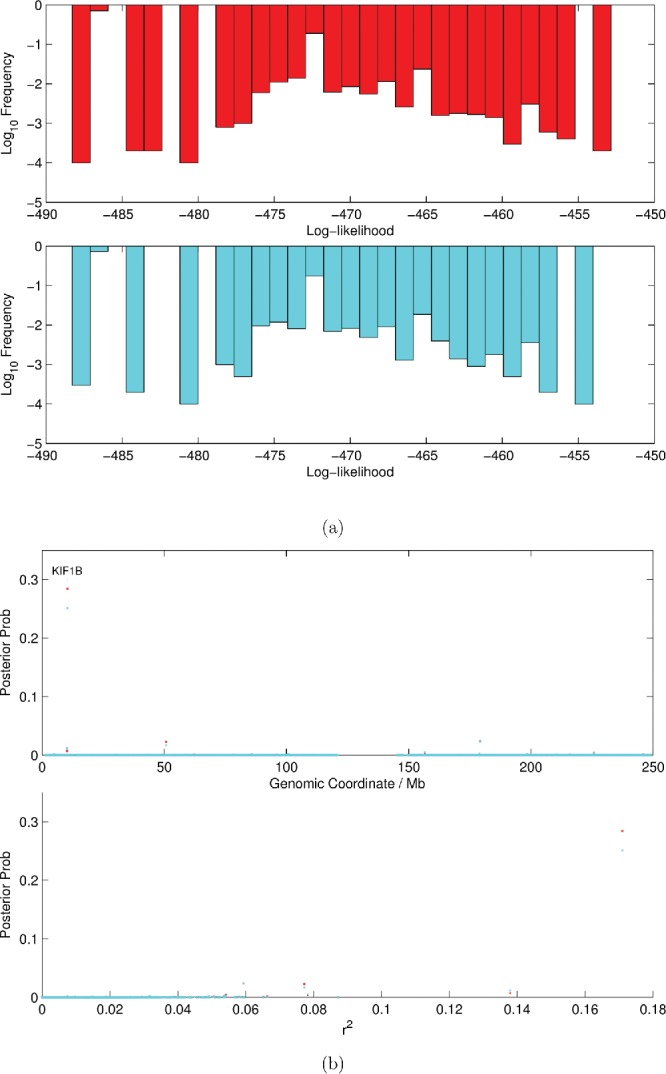
(a) Distribution of the sample log-likelihoods for the block Gibbs sampler (BG2—red) and the Hamming ball sampler (HB1—blue), respectively. (b) Estimated posterior probabilities versus genomic coordinate (top) and correlation coefficient (bottom) for the block Gibbs sampler (BG2—red) and the Hamming ball sampler (HB1—blue), respectively.

For such a high-dimensional problem, the performance of the simplest Hamming ball sampler (HB1) was particularly outstanding as it used the least CPU time and achieved a lower integrated autocorrelation time than BG1 and BG2. The performance can be explained by the fact that the Hamming ball sampling schemes can handle a large block of variables at a time but do not require exhaustive enumeration of all possible latent variable combinations within each block. This provides an important computational saving for sparse problems where most combinations will have low probability and the reason why the HB1 sampler was particularly effective for this example.

#### 3.2.4 eQTL Analysis

We tackled a real expression quantitative trait (eQTL) data analysis problem by considering a subset of the eQTL dataset from Myers et al. (2007) that consists of DNA and gene expression measurements of neuropathologically human brain samples. The full dataset corresponds to a whole-genome genotyping and expression analysis on a series of 193 neuropathologically normal human brain samples using the Affymetrix GeneChip Human Mapping 500K Array Set and Illumina HumanRefseq-8 Expression BeadChip platforms; see Myers et al. (2007) for complete details. (This dataset is freely available from *http://www.bios. unc.edu/research/genomic_software/seeQTL/data_source*, which provides a unified data depository and summary page for several genome-wide eQTL datasets.)

We specifically consider the task of discovering *cis*-associations for a certain gene, called KIF1B, on chromosome 1 so that the expression of KIF1B across samples was treated as the response in the Bayesian regression model. Local *cis*-associations had already been identified for KIF1B by Myers et al. (2007) but we sought uncover additional nonlocal associations that were still on the same chromosome. We used a subset of the dataset in Myers et al. (2007) consisting of 10,000 SNPs from chromosome 1 that we considered to be the set of covariates. These covariates were used to explain the gene expression of the gene KIF1B that exists on the same chromosome. The gene expression values of KIF1B across all samples were treated as the responses in the linear regression model. These responses were centered to have zero mean and scaled to have unit variance.

The set of 10,000 covariates was obtained from the initial large pool of 23,979 SNPs in chromosome 1 by identifying 9998 SNPs with no significant correlation with the gene expression of KIF1B (based on the empirical pairwise Pearson correlation coefficient less than 0.3). We then randomly chose two SNPs with Pearson correlation coefficient greater than 0.3 to complete the set. The goal is to identify if the samplers are able to identify one or both of these SNPs. Each covariate value for a certain SNP and sample was encoded based on three integer values in the range {0, 1, 2} so that 0 corresponds to genotype AA, 1 to genotype AB, and 2 to genotype BB. This encoding was already the data format in the data repository and it is standard for encoding SNPs in eQTL analysis.

Based on the results of the simulated data study, we tried two sampling schemes: (i) a block Gibbs sampler (BG2) and (ii) a Hamming ball sampler (HB1). These were chosen because they were sufficiently computationally inexpensive to operate on this problem involving 10,000 covariates. For both algorithms, we used a burn-in phase of 1000 iterations and then we collected 10,000 samples during the main sampling phase. Figure 4(a) shows the distribution of the log-likelihoods for both samplers. The distributions are remarkably similar demonstrating that both methods were exploring similar regions of the posterior space. However, while the block Gibbs sampler took over 26 hr to complete, the Hamming ball sampler took just 7 hr. Figure 4(b) verifies that both methods produce near-identical output in terms of the posterior probabilities estimated. Both sampling approaches identified the SNP rs12120191 (one of the two artificially included SNPs) as being a putative eQTL for KIF1B. This was also found by Myers et al. (2007) and is unsurprising since the SNP actually lies in the KIF1B coding region!

### 3.3 Tumor Deconvolution Through Mixture Modeling

We now turn to a real world application in cancer genome sequence analysis. Tumor samples are genetically heterogenous and typically contain an unknown number of distinct cell subpopulations. Current DNA sequencing technologies ordinarily produce data that come from an aggregation of these subpopulations thus, to gain insight into the latent genetic architecture, statistical modeling must be applied to deconvolve and identify the constituent cell populations and their mutation profiles.

#### 3.3.1 Setup

We assume that the data **y** = {*r_i_*, *d_i_*}^*N*^_*i* = 1_ consist of *N* pairs of read counts where *r_i_* corresponds to the number of sequence reads corresponding to the variant allele at the *i*th locus and *d_i_* is the total number of sequence reads covering the mutation site. The distribution of the variant allele reads given the total read count follows a Binomial distribution

$$\begin{array}{c} r_{i}∼\mathrm{Binomial}\left(d_{i},\varphi_{i}\right),\;i=1,\cdots ,N, \end{array}$$

where the variant allele frequency is given by ϕ_*i*_ = (1 − *e*)*p_i_* + *e*(1 − *p_i_*) and *e* is a sequence read error rate and $p_{i}=\frac{1}{2}\sum_{k=1}^{K}\theta_{k}X_{ki}.$

The parameter θ is a *K* × 1 vector denoting the proportion of the observed data sample attributed to each of the *K* tumor subpopulations whose genotypes are given by a *K* × *N* binary matrix **X**. We specify the prior probabilities over θ as

$$\begin{array}{c} \theta_{k}=\frac{\gamma_{k}}{\sum_{j=1}^{K}\gamma_{j}},\;k=1,\cdots ,K, \end{array}$$

and

$$\begin{array}{c} \gamma_{k}∼\mathrm{Gamma}\left(\alpha /K,1\right),\;k=1,\cdots ,K. \end{array}$$

This hierarchical representation is equivalent to a marginal prior distribution

$$\begin{array}{c} \theta |\alpha ∼\mathrm{Dirichlet}(\alpha /K,\cdots ,\alpha /K), \end{array}$$

which induces a sparsity forcing values of θ to be close to zero when α ⩽ 1 allowing us to do automatic model selection for the number of tumor subpopulations. The use of the auxiliary variables γ is for computational convenience and only the normalized parameters θ have a physical interpretation.

We further specify the prior probabilities over **X** as

$$\begin{array}{c} x_{ki}|f_{i}∼\mathrm{Bernoulli}\left(x_{ki},f_{i}\right),\;i=1,\cdots ,N,k=1,\cdots ,K, \end{array}$$

and

$$\begin{array}{c} f_{i}|f_{\alpha },f_{\beta }∼\mathrm{Beta}\left(f_{\alpha },f_{\beta }\right),\;i=1,\cdots ,N. \end{array}$$

This framework is similar to that recently adopted by Zare et al. (2014) and Xu et al. (2015).

#### 3.3.2 Posterior Inference

We used a Metropolis within Gibbs sampling approach incorporating our Hamming ball auxiliary variable construction to sample from the posterior *p*(θ, **X**, *f*|**y**). First, we define, *v_k_* = log (γ_*k*_), *k* = 1, …, *K*, then to update the weight parameters θ (in the implementation we actually work with *v*) we used a mixture proposal of an independent proposal drawn from the prior distribution (in this case, the Log-Gamma distribution as *v_k_* = log (γ_*k*_)) and a random walk proposal drawn from a Normal distribution centered on the current value of θ:

$$\begin{array}{c} v_{k}^{'}|v_{k}^{\left(t\right)},\sigma_{v}^{2}∼\left\{\begin{array}{cc} \text{Normal}\left(v_{k}^{\left(t\right)},\sigma_{v}^{2}\right), & \text{with}\;\text{prob.}\;\;1-\epsilon ,\\ \text{Log-Gamma}(\alpha /K,1), & \text{with}\;\text{prob.}\;\;\epsilon , \end{array}\right. \end{array}$$

The parameter ϵ is set to a small value to allow occasional proposals from the prior and potentially facilitate larger joint changes to (θ, **X**).

We accept the joint proposal using a Metropolis–Hastings step as follows:

(24) v(t+1)|y,U(t)=v', if min1,p˜(v',U(t),y)q(v(t)|v')p˜(v(t),U(t),y)q(v'|v(t))<r,v(t), otherwise ,

where the superscript *t* indicates the iteration number, *r* ∼ Uniform(0, 1) and the acceptance probability is computed according to

$$\begin{array}{c} p\left(v,U,y\right)=\sum_{X\in H_{m}\left(U\right)}\left(p\left(y|X,v\right)\left[\prod_{k=1}^{K}g\left(v_{k}|\frac{\alpha }{K},1\right)\right]\right) \end{array}$$

and *g*(*v_k_*|α/*K*, 1) is the probability density function of the log-gamma distribution with parameters (α/*K*, 1).

The above sampling move consists of a marginal Hamming ball sampler operation, as defined in Section 2.2, where together with the parameters *v* we jointly sample the full mutation matrix **X** from its restricted posterior conditional distribution, which factorizes across the columns. More precisely, the columns of **X** are considered as the blocks in the Hamming ball construction so that a new value for each column **x**_*i*_ is proposed according to the following (and accepted or rejected jointly with *v*):

(25) $$\begin{array}{ccc} & & \;\;\;\;\;\;\;\;\;\;\;\;\;\;\;\;\;\;\;\;\;\;\;\;\;\;\;\;p\left(x_{i}^{'}|y_{i},u_{i}^{\left(t\right)},\theta^{\left(t+1\right)},f_{i}^{\left(t\right)}\right)\\ & & \;\;\;\;\;\;\;\;\;\;\;\;\;\;\;\;\;\;\;\;\;\;\;\;\;\;\;\;\;=\frac{p\left(y_{i}|x_{i}^{'},\theta^{\left(t+1\right)}\right)p\left(x_{i}^{'}|f_{i}^{\left(t\right)}\right)}{\sum_{x_{i}\in H_{m}\left(u_{i}^{\left(t\right)}\right)}p\left(y_{i}|x_{i},\theta^{\left(t+1\right)}\right)p\left(x_{i}|f_{i}^{\left(t\right)}\right)}, \end{array}$$

where the normalizing constant on the denominator is truncated by the Hamming ball constraint potentially substantially reducing the computations required. For example, for (*m*, *K*) = (1, 10), the number of summation terms is 11 but for (*m*, *K*) = (3, 10) this increases to 176.

The subclonal mutation frequencies *f* are updated in a separate Gibbs step according to

(26) fi(t+1)|xi(t+1),fα,fβ∼ Beta fα+∑k=1KIxki(t+1)=1,fβ+K-∑k=1KIxki(t+1)=1,

where *I*( · ) denote the indicator function. Finally, the Hamming ball auxiliary variables **U** are updated column-wise by sampling uniformly from the Hamming ball centered on each column of the mutation matrix **x**_*i*_ according to the distribution in (8).

In all simulations, we use a setting of ϵ = 0.01 for the mixture proposal. We used a burn-in phase of 10,000 iterations (including an initial 1000 iteration tuning phase) and took 100,000 samples during the main sampling run. We calibrated the variance of the proposal distribution σ^2^_*v*_ during the tuning phase to achieve an acceptance rate of between 10% and 40% but disallowed the variance from falling below 0.01 or going above 10.

We compared three posterior sampling approaches: (i) our Hamming ball-based sampling scheme as defined above, (ii) a conventional block Gibbs sampling strategy that proceeds by conditionally updating one column **x**_*i*_ at a time with the remaining columns **X**_− *i*_ and the weights θ fixed, and (iii) a *fully marginalized* sampling strategy where **X** was marginalized through exhaustive summation over all column configurations (note, this corresponds to the Hamming ball sampler with *m* = *K*).

#### 3.3.3 Results

**Figure 5.**
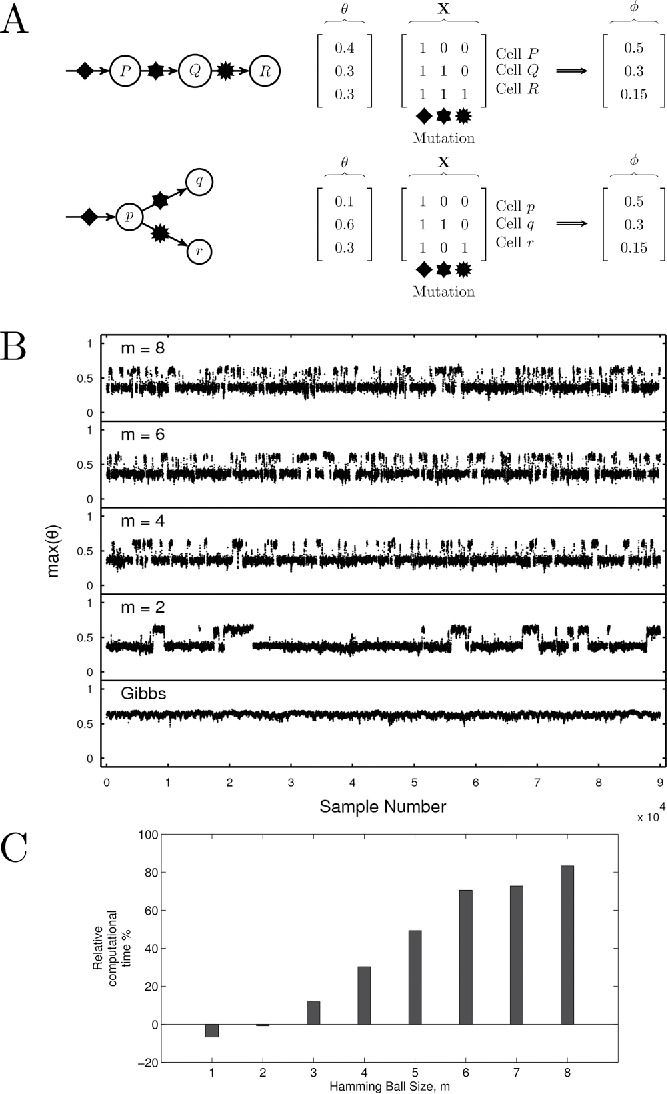
Tumor deconvolution. (a) Two distinct clonal architectures that lead to the same mutant allele frequency vector ϕ = [0.5, 0.3, 0.15]′. (b) Trace plots showing the sampled values of max (θ). (c) Relative computational times for the Hamming ball sampler for various *m* (times relative to the block Gibbs sampler).

For the simulation study, we considered a simulated data example, illustrated in Figure 5(a), where the observed sequence data are generated so that it can be equally explained by two different latent genetic architectures. This is an interesting example as one configuration corresponds to a linear phylogenetic relationship between cell types and the other to a branched phylogeny and represent fundamentally different evolutionary pathways. For this example, we would expect an *efficient* sampler to identify both configurations and to be able to move freely between the two during the simulation revealing the possibility of the existence of dual physical explanations for the observed data.

We simulated data by forward simulation from this assumed system. Specifically, we assumed the sequencing read depth *d* = 800, which represents a high coverage typical of a target resequencing experiment (note that our objective here is to explore the properties of the inference algorithm as supposed to the accuracy of the generative model or the experimental designs. Therefore, we have not exhaustively explored the interactions between these factors, which will be the subject of future investigation) and the following parameters:

$$\begin{array}{c} \theta_{0}=\left(\begin{array}{c} 0.3\\ 0.3\\ 0.4 \end{array}\right),\;X_{0}^{'}=\left(\begin{array}{ccccccccc} 1 & 1 & 1 & 1 & 1 & 1 & 1 & 1 & 1\\ 1 & 1 & 1 & 1 & 1 & 1 & 0 & 0 & 0\\ 1 & 1 & 1 & 0 & 0 & 0 & 0 & 0 & 0 \end{array}\right), \end{array}$$

where **X**′_0_ is a version of **X**_0_ shown in Figure 5(a) with replicated columns, *r_i_* ∼ Binomial(d, p_0i_), i = 1, …, 9, where *p*_0_ = θ^*T*^_0_ **X**′_0_ to give values of

$$\begin{array}{c} r={\left[405,397,393,239,245,247,123,121,123\right]}^{'}. \end{array}$$

We chose hyperparameter values of α = 1, *f*_0_ = 0.5, (*f*_α_, *f*_β_) = (0.5, 0.5), and *e* = 10^− 2^.

Figure 5(b) and 5(c) displays trace plots of the largest component weight, max (θ), and the relative computational times for the three sampling schemes. We use max (θ) as meaningful visualization of the multidimensional mutation matrix **X** is challenging. All Hamming ball samplers were effective at identifying both modes but the efficiency of the mode switching depends on the Hamming ball size *m*. This effectiveness can be attributed to the fact that the Hamming ball schemes can *jointly* propose to change up to 2*mN* bits across *all* *N* columns of the current **X**. Furthermore, conditional updates of θ can be made by marginalizing over a range of mutation matrices. In fact, for *m* ⩾ *K*/2, each iteration of the Hamming ball sampler allows any element of **X** to be changed but, unlike the fully marginalized sampling procedure (*m* = *K* = 8), it is more computationally tractable if the number of mutations is large as exhaustive enumeration is not required. The conditional updates employed by the block Gibbs sampler require significantly less computational effort but the approach is prone to being trapped in single posterior mode and our simulations show that it failed to identify the mode corresponding to the linear phylogeny structure (max θ = 0.4).

#### 3.3.4 Real Data Analysis

We next sought to[Fig f0006]demonstrate the method using real mutation sequencing data. We extended the model to allow each tumor sample *s* to have its own mixture weights θ^(*s*)^ over a common pool of *K* possible cell types defined by **X** and performed MCMC inference over the joint distribution *p*(θ^(1)^, …, θ^(13)^, **X**|**y** ^(1)^, …, **y** ^(13)^).

We obtained the breast cancer data from Zare et al. (2014). This dataset consists of 17 confirmed somatic variants sequenced in 12 tumor samples and 1 normal tissue sample obtained from a single breast cancer patient (10 samples from 3 primary sections and 2 samples from a metastasis). As in Zare et al. (2014), we modified the model and inference algorithm to account for the multiple datasets by assuming that the data for each tumor sample are generated from a common mutation matrix **X** but each tumor sample *s* ∈ {1, …, 12} is associated with its own weight parameters θ_*s*_.

**Figure 6. f0006:**
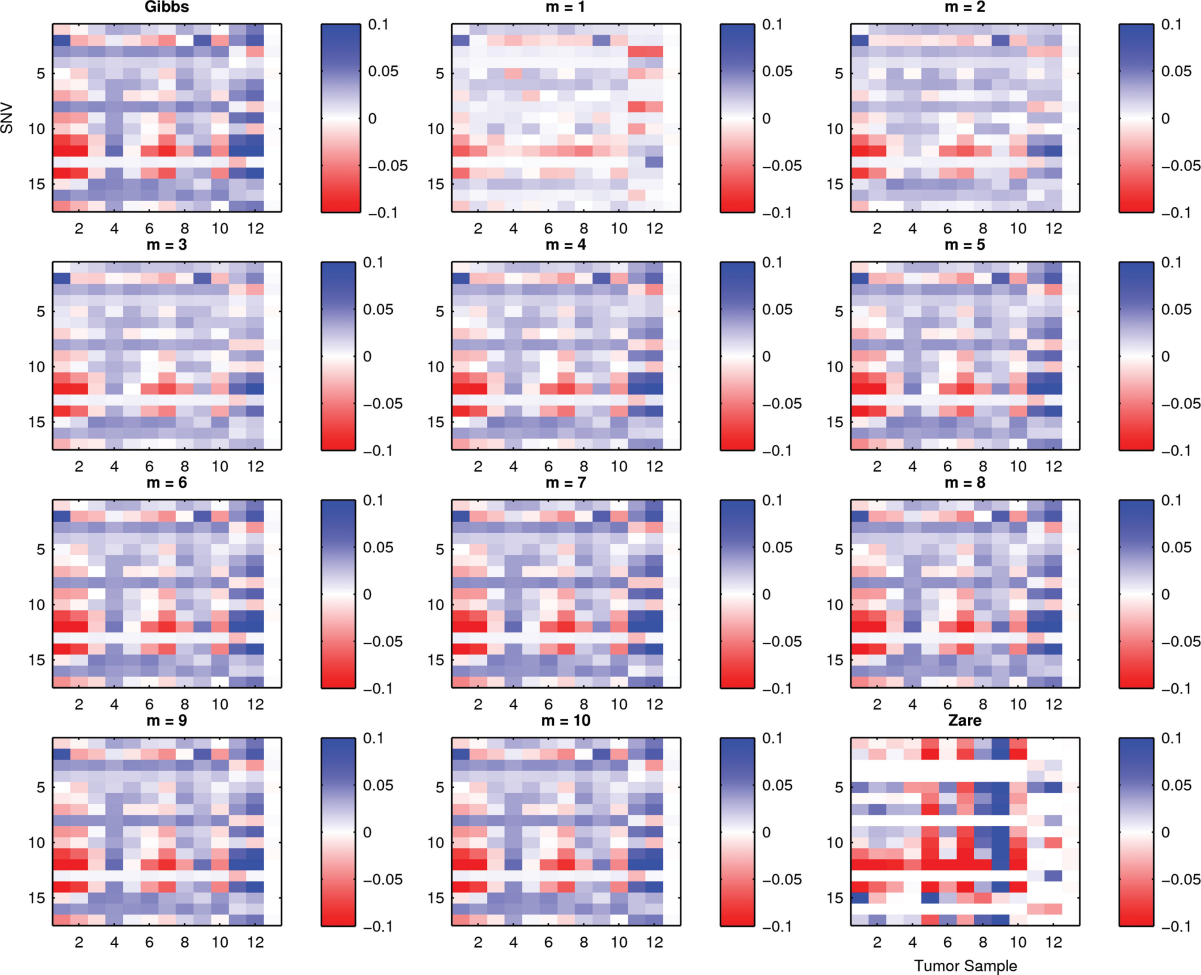
Breast cancer model fit. Heatmaps showing the discrepancy between the posterior mean of the variant allele frequencies ϕ for each mutation and tumor sample against the observed variant allele frequency *r*/*d*. Model fits are shown for the block Gibbs sampler, the Hamming ball sampler (for various *m*) and reported results from Zare et al. (2014) (for their most complex model involving up to six sub-clones).

Figure 6 shows heatmaps of the model fit residuals calculated as

$$\begin{array}{c} {\text{Residual}}_{ij}=\text{Observed}\;\text{VAF}r_{ij}/d_{ij}-\text{Posterior}\;\text{mean}\;\text{of}\;\varphi_{ij} \end{array}$$

for the *i*th mutation of the *j*th sample for each of the sampling-based algorithms and compared to the six-component model given in Zare et al. (2014). In all instances, the Bayesian model fits are superior to the point estimates given by the EM algorithm of Zare et al. (2014), which shows some extreme discrepancies and there is little difference between the block Gibbs and Hamming ball samplers.

**Figure 7.**
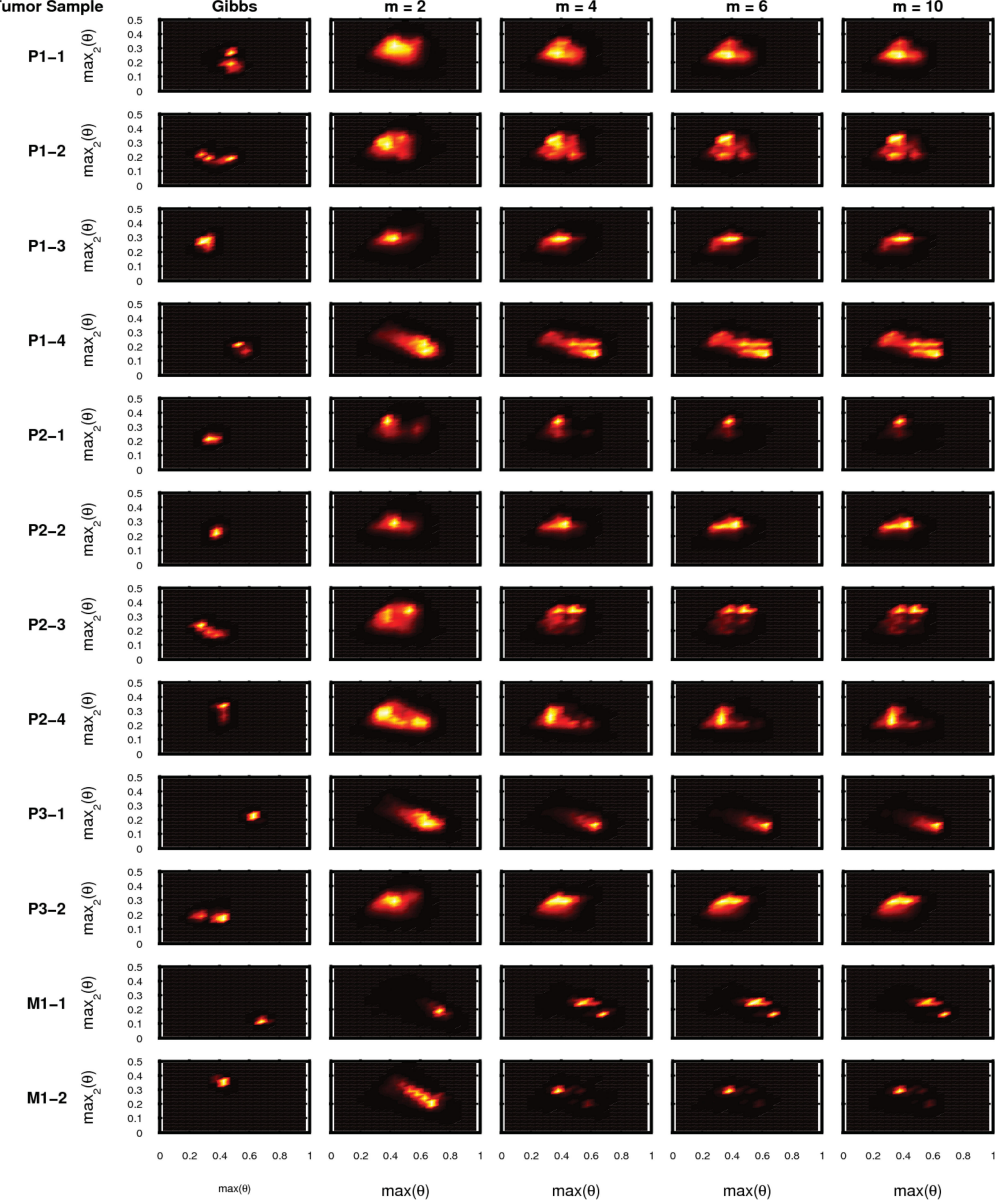
Breast cancer model fit. Posterior distributions of maximum two values of θ for each tumor sample for each of the sampling algorithms.

Figure 7 shows the posterior distribution of the largest two values of θ for each tumor sample given by each of the samplers. The posterior distributions given by the Hamming ball sampler tend to exhibit greater variance and captures multiple modes demonstrating that it is exploring the posterior model space better than the block Gibbs sampler. In fact, the posterior distribution given by the block Gibbs sampler tends to be highly concentrated in relatively small areas of the posterior space but this area of the space cannot correspond to the only values of (θ, **X**) consistent with the data as the Hamming ball sampler is able to explore a greater range of parameter values that give residual fits as good as the block Gibbs sampler (see Figure 6). In combination, the two results show that there are indeed a large range of possible model configurations that can give rise to the same observed data and that the Hamming ball sampler better captures the true statistical uncertainty than the point estimates of Zare et al. (2014) or a standard block Gibbs sampler. Note that in this case a Hamming distance *m* = 4 gives approximately the same result as *m* = 10 (the exact marginal sampling approach) but with a substantial computational saving.

Overall, we demonstrate two important points. First, misleading inferences can be made about the latent subclonal architecture of a tumor not only through poor data quality or model misspecification but also an inadequate inference procedure. In this case, the use of conditional updates in a standard block Gibbs sampling approach is inappropriate due to correlations between θ and **X**. Second, the statistically most efficient marginal sampling scheme is prohibitively expensive to run for large problems. Our Hamming ball sampling strategies provides access to a continuum of intermediate sampling schemes that lie between the extremes offered by the block Gibbs and marginal approaches. This gives control *to the analyst* to determine a suitable inferential procedure that is appropriate for *their* resources and time frame.

## 4. Discussion

### 4.1 Related Methods

The Hamming ball sampler provides a generic sampling scheme for statistical models involving high-dimensional discrete latent state-spaces that generalizes and extends conventional block Gibbs sampling approaches. It can be considered as a type of slice sampling that is suitable for discrete combinatorial state spaces. It differs from regular slice samplers that are based on uniform sampling in the subgraph of the probability distribution (see Robert and Casella (2005) for a review), since now the model slicing is constructed directly around a local neighborhood set centered on an auxiliary variable that is resampled based also on the same neighborhood set principle. Furthermore, the slicing idea in Hamming ball sampling leads to a computationally tractable algorithm for discrete state spaces while for continuous high-dimensional spaces it would be intractable. To see this, notice that if we were to slice based on the set {**U**: ||**U** − **X**|| ⩽ *m*} where $U,X\in R^{D}$ and || · || denotes a continuous norm, the step of sampling **X** would require simulating from a truncated high-dimensional continuous distribution, which is typically infeasible.

The neighborhood approach used by the Hamming ball sampler resembles certain types of purely M-H schemes, particularly the Metropolized Shotgun Stochastic Search (M-SSS) procedure (see sec. 4.1 in Hans, Dobra, and West 2007) that has been applied in sparse linear regression. More precisely, M-SSS can be viewed as using the restricted space defined by the Hamming Ball set $H_{m}\left(X\right)$ (where radius was set to *m* = 1 in Hans, Dobra, and West 2007) to construct directly a proposal distribution in a accept/reject M-H step to sample **X** without the use of the intermediate auxiliary variable **U**. Specifically, if **X** ^(*t*)^ is the value of the current state we can define a proposal *Q*(**X**′|**X** ^(*t*)^) by slicing the exact model probability distribution around **X** ^(*t*)^ according to

(27) $$Q\left(X^{'}|X^{\left(t\right)}\right)=\frac{p\left(y,X^{'},\theta \right)I\left(X^{'}\in H_{m}\left(X^{\left(t\right)}\right)\right)}{Z_{m}\left(X^{\left(t\right)}\right)},$$

where $Z_{m}\left(X^{\left(t\right)}\right)=\sum_{X^{'}\in H_{m}\left(X^{\left(t\right)}\right)}p\left(y,X^{'},\theta \right)$. Sampling then proceeds by proposing a certain **X**′ from the above instrumental distribution, which is then accepted (i.e., the next state is set to **X** ^(*t* + 1)^ = **X**′) with probability

(28) $$\begin{array}{ccc} & & \min\left(1,\frac{p(y,X^{'},\theta )}{p(y,X^{\left(t\right)},\theta )}\frac{\frac{p\left(y,X^{\left(t\right)},\theta \right)I\left(X^{\left(t\right)}\in H_{m}\left(X^{'}\right)\right)}{Z_{m}\left(X^{'}\right)}}{\frac{p\left(y,X^{'},\theta \right)I\left(X^{'}\in H_{m}\left(X^{\left(t\right)}\right)\right)}{Z_{m}\left(X^{\left(t\right)}\right)}}\right)\\ & & \;=\min\left(1,\frac{Z_{m}\left(X^{\left(t\right)}\right)}{Z_{m}\left(X^{'}\right)}\right), \end{array}$$

otherwise **X**′ is rejected and **X** ^(*t* + 1)^ = **X** ^(*t*)^. Notice that *Z_m_*(**X** ^(*t*)^) is the probability mass or volume of the sliced part of the model around **X** ^(*t*)^, which is the state we are starting from, while similarly *Z_m_*(**X**′) is the volume around the state we attempt to move into. Thus, to satisfy detailed balance the above M-H probability needs to take into consideration the probability volume around the current state and the volume around the proposed state.

The above scheme has two main differences with the Hamming ball sampler. First, controlling the acceptance rate in the above scheme, when sampling **X**, is difficult and sampling might exhibit unpredictably low acceptance rate so that the chain can get stuck to the same state for many iterations. In contrast, the Hamming ball sampler has exactly the same computational cost with the above pure M-H algorithm, but through the specific auxiliary variable construction we can derive a Gibbs sampler that always accepts any proposed **X** ^(*t* + 1)^.

A second important difference is that the amount of possible exploration (per iteration) based on the above purely M-H scheme is half the amount of exploration of the Hamming ball sampler for the same value of the radius *m* and the same computational cost. This is because now the next state **X** ^(*t* + 1)^ can differ from the current **X** ^(*t*)^ in at most *mP* elements. In contrast, in the Hamming ball sampler the next (always accepted) **X** ^(*t* + 1)^ can differ from **X** ^(*t*)^ by up to 2*mP* elements.

### 4.2 Extensions of the Hamming Ball Sampler

A generalization of the algorithm is obtained by allowing block-wise random maximal Hamming distances.

More precisely, if we assume a varying radius for the individual Hamming balls, then the conditional distribution over **U** is now uniform on the generalized Hamming ball $H_{m}\left(X\right)=\left\{U:d\left(u_{i},x_{i}\right)\leq m_{i},i=1,\ldots ,P\right\},$ where **m** = (*m*_1_, …, *m_P_*) denotes the set of maximal distances for each subset of variables. Furthermore, we can allow **m**, at each iteration, to be randomly drawn from a distribution *p*(**m**), which leads us to the following generalization of the algorithm:

(29) (U,m)←p(U|X,m)p(m),[3pt]

(30) (θ,X)←p(θ,X|y,U,m),

where again the second step can be implemented either by applying two conditional Gibbs steps or a joint M-H step. This scheme remains valid since essentially it is Gibbs sampling in an augmented probability model where we added the auxiliary variables (*U*, **m**).

Furthermore, via the randomness over **m** we can again interpret standard block Gibbs Samplers as special cases. When *p*(**m**) is such that at each iteration it randomly picks a single index *i*, sets $m_{i}=\text{length}\left(x_{i}\right)$ and for the rest *j* ≠ *i* sets *m_j_* = 0, the algorithm reduces to a block Gibbs sampler. Since, in such a case, **x**_*i*_ would be freely sampled from its conditional *p*(**x**_*i*_|**X**_− *i*_, θ, **y**) while the remaining blocks **X**_− *i*_ = {**x**_*j*_: *j* ≠ *i*} would be frozen to their current values because the maximal distances of their individual Hamming balls are zero.

In practice, using a varying or random **m** could lead to more efficient variations of the Hamming ball sampler where, for instance, the vector **m** could be automatically tuned during sampling to focus computational effort in regions of the sequence where there is most uncertainty in the underlying latent structure of **X**.

A further extension can be obtained by using a more complex form for the auxiliary conditional distribution *p*(**U**|**X**). For instance, a simple generalization is to assume an auxiliary distribution of the form

(31) $$p\left(U|X\right)=\prod_{i=1}^{P}\frac{1}{Z_{i,m}}\exp\left(-\lambda d\left(u_{i},x_{i}\right)\right)I\left(u_{i}\in H_{m}\left(x_{i}\right)\right),$$

where the parameter λ ⩾ 0 controls the variance of the distribution, When λ = 0, the above reduces to the uniform distribution while as λ increases *p*(**U**|**X**) places more and more probability mass toward the center **X** in a spherically symmetric way. Given that all vector **x**_*i*_’s have size *K*, the overall normalizing constant can be written as *Z_m_* = *M^P^*_λ_ where Mλ=∑j=0me-λj(S-1)jKj, which is a weighted volume of each block-specific Hamming ball that still remains independent from **x**_*i*_. Notice that in the above scheme λ is a parameter that the user could tune to optimize sampling performance.

Other more complex forms of the auxiliary distribution could be possible (for instance, this distribution could depend on the data **y**). However, from practical point of view, it is critical that this distribution factorizes across the blocks, similarly to (31), and it has an analytically computed normalizing constant so that exact simulation is straightforward. In our simulations, we experiment with the simplest (and perhaps the most practical) Hamming ball schemes where the Hamming radius is fixed and the auxiliary distribution is uniform and leave the above more elaborate cases for future work.

## 5. Conclusions

In our investigations, we have applied the Hamming ball sampling scheme to three different statistical models and shown benefits over standard Gibbs samplers. Importantly, the Hamming ball sampler gives the statistical investigator control over the balance between statistical efficiency and computational tractability through an intuitive mechanism—the specification of the Hamming Ball radius and the block design strategy—which is important for Big Data applications where the volume of data precludes exact analysis. Yet, we have also demonstrated that in many problems, basic Hamming ball samplers (*m* = 1 or *m* = 2) that are computationally inexpensive can still give relatively good performance compared to standard block Gibbs sampling alternatives.

Throughout we have provided pure and unrefined Hamming ball sampler implementations. In actual applications, the proposed methodology should not be seen as a single universal method for speeding up MCMC but a novel addition to the toolbox that is currently available to us. For example, the computations performed within each Hamming ball update are often trivially parallelizable, which would allow the user to take advantage of any special hardware for parallel computations, such as graphics processing units (Lee et al. 2010; Suchard et al. 2010). In addition, Hamming ball sampling updates can also be used alongside standard Gibbs sampling updates as well as within parallel tempering schemes in Evolutionary Monte Carlo algorithms (Brooks et al. 2011).

Finally, we believe the ideas presented here can have applications in many areas not yet explored, such as Bayesian nonparametrics (e.g., in the Indian Buffet Process; Griffiths and Ghahramani 2005) and Markov random fields. Further investigations are being conducted to develop the methodology for these statistical models.

## Supplementary Material

Supplementary Materials

## References

[cit0001] Bottolo L., and Richardson S. (2010), “Evolutionary Stochastic Search for Bayesian Model Exploration,” *Bayesian Analysis*, 5, 583–618.

[cit0002] Brooks S., Gelman A., Jones G., and Meng X.-L. (2011), *Handbook of Markov Chain Monte Carlo*, Boca Raton, FL: CRC Press.

[cit0003] Griffiths T. L., and Ghahramani Z. (2005), “Infinite Latent Feature Models and the Indian Buffet Process,” *NIPS*, 18, 475–482.

[cit0004] Hans C., Dobra A., and West M. (2007), “Shotgun Stochastic Search for ‘large P’ Regression,” *Journal of the American Statistical Association*, 102, 507–516.

[cit0005] Lee A., Yau C., Giles M. B., Doucet A., and Holmes C. C. (2010), “On the Utility of Graphics Cards to Perform Massively Parallel Simulation of Advanced Monte Carlo Methods,” *Journal of Computational and Graphical Statistics*, 19, 769–789. 22003276 10.1198/jcgs.2010.10039 PMC3191530

[cit0006] Myers A. J., Gibbs J. R., Webster J. A., Rohrer K., Zhao A., Marlowe L., Kaleem M., Leung D., Bryden L., Nath P., Zismann V. L., Joshipura K., Huentelman M. J., Hu-Lince D., Coon K. D., Craig D. W., Pearson J. V., Holmans P., Heward C. B., Reiman E. M., Stephan D., and Hardy J. (2007), “A Survey of Genetic Human Cortical Gene Expression,” *Nature Genetics*, 39, 1494–1499. 17982457 10.1038/ng.2007.16

[cit0007] O’Hara R. B., and Sillanpää M. J. (2009), “A Review of Bayesian Variable Selection Methods: What, How and Which,” *Bayesian Analysis*, 4, 85–117.

[cit0008] Peltola T., Marttinen P., and Vehtari A. (2012), “Finite Adaptation and Multistep Moves in the Metropolis-Hastings Algorithm for Variable Selection in Genome-Wide Association Analysis,” *PloS One*, 7, e49445. 23166669 10.1371/journal.pone.0049445 PMC3499564

[cit0009] Robert C. P., and Casella G. (2005), *Monte Carlo Statistical Methods (Springer Texts in Statistics)*, Secaucus, NJ: Springer-Verlag New York, Inc.

[cit0010] Schäfer C., and Chopin N. (2013), “Sequential Monte Carlo on Large Binary Sampling Spaces,” *Statistics and Computing*, 23, 163–184.

[cit0011] Suchard M. A., Wang Q., Chan C., Frelinger J., Cron A., and West M. (2010), “Understanding GPU Programming for Statistical Computation: Studies in Massively Parallel Massive Mixtures,” *Journal of Computational and Graphical Statistics*, 19, 419–438. 20877443 10.1198/jcgs.2010.10016 PMC2945379

[cit0012] Swendsen R. H., and Wang J.-S. (1987), “Nonuniversal Critical Dynamics in Monte Carlo Simulations,” *Physical Review Letters*, 58, 86– 88. 10034599 10.1103/PhysRevLett.58.86

[cit0013] Xu Y., Müller P., Yuan Y., Gulukota K., and Ji Y. (2015), “MAD Bayes for Tumor Heterogeneity–Feature Allocation With Exponential Family Sampling,” *Journal of the American Statistical Association*, 110, 503–514. 26170513 10.1080/01621459.2014.995794 PMC4498588

[cit0014] Zare H., Wang J., Hu A., Weber K., Smith J., Nickerson D., Song C., Witten D., Blau C. A., and Noble W. S. (2014), “Inferring Clonal Composition From Multiple Sections of a Breast Cancer,” *PLoS Computational Biology*, 10, e1003703. 25010360 10.1371/journal.pcbi.1003703 PMC4091710

[cit0015] Zellner A. (1986), “On Assessing Prior Distributions and Bayesian Regression Analysis With **g**-Prior Distributions,” in *Bayesian Inference and Decision Techniques-Essays in Honour of Bruno de Finetti*, eds. P. Goel and A. Zellner, New York: Elsevier, pp. 233–243.

