# Supplementary material for: The Hamming Ball Sampler
Source: J Am Stat Assoc. 2017 Sep 3;112(520):1598–611. doi: 10.1080/01621459.2016.1222288 (PMC5796496; doi:10.1080/01621459.2016.1222288)
Supplement: Supplementary Materials [file UASA_A_1222288_SM9424.pdf]

Supplementary Information

Contents

1 Blocking strategies **1**

2 Factorial hidden Markov models **2**

List of Figures

1 Sampling from a posterior distribution over $D = 14$ binary variables having two symmetric modes so that jumping between the modes requires simultaneously flipping two certain binary variables. The left panel shows the computational complexity for all block Hamming Ball schemes. The middle panel shows the corresponding sampling efficiency which was defined as the transition probability between the two modes (an ideal exact sampler has maximum transition probability equal to 0.5). Finally, the right panel displays the ratio between sampling efficiency and computational complexity which is maximized for $(m, K) = (1, 13)$. For all panels brighter colours indicate higher values. . . . 6

2 Performance in the training phase for the REDD dataset. Panel (a) shows the actual CPU times for carrying out 10,000 sampling iterations for fitting the FHMM to a sequence of length $9,600$ electricity measurements. Panel (b) shows the evolution of the log joint density while panel (c) gives the evolution of the MSE scores. 7

3 Performance of the prediction phase for the REDD dataset when each algorithm uses its own most representative sample over $(\mathbf{W}, \sigma^2)$ taken to be the last sample after 10,000 iterations in the training phase (panel b) and by using a common representative sample for all algorithms taken to be the last sample from HB2 (panel c). The panel in (a) shows the actual CPU times for carrying out 300 sampling iterations for analyzing the full test sequence of length 57,600. 8

4 Performance on the 7 day REDD data set. Each row displays the actual values of the electricity measurements in the REDD dataset (black dots) together with the mean prediction/reconstruction found by the HB2 algorithm (blue or red). Specifically, the panel in the first row shows the reconstruction in the training sequence, corresponding to Day 1, that was considered for training the FHMM, while the remaining 6 panels show the reconstructions of the full test sequence broken down to individual days. 9

1 Blocking strategies

Here, we further discuss the use of Hamming Ball sampling, and its connection with Gibbs sampling, for the case $\mathbf{X}$ is just a large unstructured D-dimensional vector so that we iteratively apply conditional Hamming Ball moves to random blocks of $\mathbf{X}$. Notice that such algorithm was used in the simulated sparse linear regression example and the real eQTL dataset.

At each iteration we divide the $\mathbf{X}$ variables into randomly chosen blocks $\mathbf{x}_i$, $i = 1, \ldots, P$, so that they have common length $K = \text{length}(\mathbf{x}_i)$[1]. However, instead of sampling each block conditional on the others using standard block Gibbs sampling (which will be infeasible when the block size is large), we use the Hamming Ball operation, i.e. $\mathbf{u}_i \leftarrow p(\mathbf{u}_i|\mathbf{x}_i)$ and $\mathbf{x}_i \leftarrow p(\mathbf{x}_i|\mathbf{X}_{-i}, \theta, \mathbf{u}_i, \mathbf{y})$, and where the radius of the each Hamming Ball is m. All different

[1] If D/P is not a integer, then the final block $\mathbf{x}_P$ will have size smaller than K. One way to deal with such case is to expand this block to make it have size K by including some variables of the first block $\mathbf{x}_1$.

such schemes can be expressed by varying the block size $K \in \{1, \ldots, D\}$ and the radius $m \in \{1, \ldots, K\}$. Also these schemes can be visualized by a $D \times D$ upper triangular matrix where rows represent the radii and the columns the block sizes. The diagonal elements of this matrix, where $m = K$, are precisely all standard block Gibbs Samplers, while the off-diagonal elements, where $m < K$, are block Hamming Ball schemes.

The most effective block Hamming Ball scheme can correspond to a certain pair (m, K) which shall trade off between computational time and sampling efficiency. The computational time is dominated by the number of operations needed to evaluate each conditional $p(\mathbf{x}_i|\mathbf{X}_{-i}, \theta, \mathbf{u}_i, \mathbf{y})$ for $i = 1, \ldots, P$. We can easily see that this involves $M \times P$ operations where M is the volume of the individual Hamming Ball set $\mathcal{H}_m(\mathbf{x}_i)$ and $P = D/K$ is the number of blocks. On the other hand, the sampling efficiency characterizes the sampler in terms of its ability to explore the probability distribution and jump between modes. Sampling efficiency depends also on the landscape and correlation structure of the probability distribution. For instance, if the posterior distribution $p(\mathbf{X}|\theta, \mathbf{y})$ factorizes across all sites in $\mathbf{X}$ then any standard block Gibbs scheme with $m = K$, $m = 1, \ldots, D$, will draw exact independent samples from the conditional posterior distribution over $\mathbf{X}$ and therefore it will have maximum sampling efficiency. However, the fastest scheme will be the one with $m = K = 1$ since it will have the smallest computational time, and hence we can say that it has the maximum *overall efficiency*. For correlated posterior distributions the overall efficiency could be maximized by some pair (m, K) that might not correspond to a standard block Gibbs Sampler. For instance, Figure 1 shows an example in variable selection in regression (generated exactly analogously as the example used in the *Results* section of the main paper and described further in Section 3.2) where $\mathbf{X}$ is a binary vector of size $D = 14$ and the posterior distribution has two completely symmetric modes so that jumping between modes requires simultaneously flipping the values of two specific variables. The right panel in Figure 1 shows the overall efficiency, defined here as the transition probability between the two modes over the time complexity, which is maximized for $(m, K) = (1, 13)$.

2 Factorial hidden Markov models

Here, we consider a real-world example from the field of energy disaggregation where the objective is to determine the component devices from an aggregated electricity signal. This technology is useful because having a decomposition, into components for each device, of the total electricity usage in a household or building can be very informative to consumers and increase awareness of energy consumption which subsequently can lead to possibly energy savings. For full details regarding the energy disaggregation application see [1, 2, 3]. Next we consider a publicly available data set[2], called the Reference Energy Disaggregation Data Set (REDD) [2], to test the HB and BG sampling algorithms. The REDD data set contains several types of home electricity data for many different houses recorded during several weeks. Next, we will consider the main signal power of `house_1` for seven days which is a temporal signal of length $604,800$ since power was recorded every second. We further downsampled this signal to every 9 seconds to obtain a sequence of $67,200$ size in which we applied the FHMM described below.

Energy disaggregation can be naturally tackled by an additive FHMM framework, as realized in [1, 2], where an observed total electricity power y_i at time instant i is the sum of individual powers for all devices that are "on" at that time. Therefore, the observation model for the additive FHMM (see eq. 33 in the main article) can be used to model this situation with the constraint that each device contribution w_k (which is now a scalar) is restricted to be non-negative. We assume an FHMM with $K = 10$ factors and we follow a Bayesian framework where each w_k is parametrized by the exponential transformation, i.e. $w_k = e^{\widetilde{w}_k}$, and a vague zero-mean Gaussian prior is assigned on $\widetilde{w}_k$ so that $\widetilde{w}_k \sim \mathcal{N}(0, 100)$. To learn these factors we apply unsupervised learning using as training data the first day of recorded data, which consist of a $9,600$ observations. This involves applying an Metropolis-within-Gibbs

[2]Available from `http://redd.csail.mit.edu/`.

type of MCMC algorithm that iterates between the following three steps: i) sampling $\mathbf{X}$, ii) sampling each $\widetilde{w}_k$ individually using its own Gaussian proposal distribution and accepting or rejecting based on the M-H step and iii) sampling the noise variance σ^2 based on its conjugate inverse Gamma posterior distribution. Notice that the step ii) involves adapting the variance of the Gaussian proposal to achieve an acceptance ratio between 20 and 40 percent following standard ideas from adaptive MCMC. We consider one of the following four algorithms: BG1, BG2, HB1 and HB2 defined in Section 5.3 in the main article. Once the FHMM has been trained then we would like to do predictions and infer the posterior distribution over the hidden factors for a test sequence, that will consist of the remaining six days, according to

$$p(\mathbf{X}_*|\mathbf{y}_*, \mathbf{y}) = \int p(\mathbf{X}_*|\mathbf{y}_*, \mathbf{W}, \sigma^2) p(\mathbf{W}, \sigma^2|\mathbf{y}) d\mathbf{W} d\sigma^2 \approx \frac{1}{T} \sum_{t=1}^{T} p(\mathbf{X}_*|\mathbf{y}_*, \mathbf{W}^{(t)}, (\sigma^2)^{(t)}), \tag{1}$$

where $\mathbf{y}_*$ denotes the test observations, $\mathbf{W} = (w_0, \ldots, w_K)$ and $\mathbf{X}_*$ the corresponding hidden sequence we wish to infer.[3] This computation requires to be able to simulate from $p(\mathbf{X}_*|\mathbf{y}_*, \mathbf{W}, \sigma^2)$ for a given fixed setting for the parameters $(\mathbf{W}, \sigma^2)$. Such a prediction step can tell us which factors are "on" at each time. Such factors could directly correspond to devices in the household, such as Electronics, Lighting, Refrigerator etc, however since our learning approach is purely unsupervised we will not attempt to establish correspondences between the inferred factors and the household appliances and, instead, we will focus on comparing the ability of the sampling algorithms to escape from local modes of the posterior distribution. To quantify such ability we will consider the mean squared error (MSE) between the model mean predictions and the actual data. Clearly, MSE for the test data can measure how well the model predicts novel electricity powers, while MSE at the training phase can indicate how well the chain mixes and reaches areas with high probability mass (where training data are reconstructed with small error). For the training phase we will also consider the log joint density (see eq. 32 in the main article) as a further indication of the ability of sampling from areas with high probability. Figure 2 shows the CPU times, the evolution of the log joint density and the MSE for the four MCMC algorithms used for training. Clearly, the HB2 algorithm is mixing better than the remaining algorithms (e.g. it reaches higher joint probability values) and at the same time it has comparable CPU time with the fastest algorithm which is HB1. Notice that the MSE curve for HB2 eventually reaches a level of values that fluctuate very close to zero (which corresponds to very efficient reconstruction of the energy powers) which is an indication that the posterior mode discovered by HB2 is possibly the most representative mode of the posterior distribution. In contrast, the BG schemes have a great tendency to get stuck to local modes from which they are very unlikely to escape. Notice, however, that BG2, that considers more global moves since it jointly samples pairs of rows in $\mathbf{X}$, reaches a local posterior mode with higher joint probability values and smaller MSE values than the BG1 scheme.

Figure 3 shows the CPU times (panel a) and the MSE curves (panel b) for the prediction phase, i.e. when sampling from $p(\mathbf{X}_*|\mathbf{y}_*, \mathbf{W}, \sigma^2)$ given a representative sample from the posterior $p(\mathbf{W}, \sigma^2|\mathbf{y})$ over the model parameters. As a most representative sample we used the final sample (from the 10,000 iterations in total) produced by the four algorithms from the training phase, so that each algorithm used its own last sample for $(\mathbf{W}, \sigma^2)$. In addition, to further examine the difference between the algorithms in terms of their ability to purely sample $\mathbf{X}$, we also repeated the prediction phase by assuming a common representative sample $(\mathbf{W}, \sigma^2)$ from the posterior distribution. Given that HB2 mixes best (see Figure 2) we chosen this common representative sample to the last sample produced by HB2. Clearly, under this second implementation of the prediction phase any difference observed between the algorithms shall depend solely on their ability to sample from $p(\mathbf{X}_*|\mathbf{y}_*, \mathbf{W}, \sigma^2)$. Figure 3(c) shows the MSE curves which again indicate the superiority of the HB2 scheme. More precisely, in this case the behaviour of the schemes

[3] Notice that we have also assumed that the training and test sequences are conditionally independent given the model parameters $(\mathbf{W}, \sigma^2)$.

BG1-2 and HB1 has significantly improved compared to the prediction based on their own mostly representative sample (Figure 3(b)). This indicates that HB2 was really more effective in sampling more representative values (during the training phase) for the mean energy parameters $(w_1, \ldots, w_K)$. Notice also that in this case HB1 outperforms both BG algorithms while it is outperformed by the best HB2 scheme.

Finally, Figure 4 gives illustrative plots on how the FHMM fits the data for all seven days so that each row corresponds to a full day of electricity powers. The plots in this figure were produced based on the HB2 output.

References

[1] Hyungsul Kim, Manish Marwah, Martin F. Arlitt, Geoff Lyon, and Jiawei Han. Unsupervised disaggregation of low frequency power measurements. In *SDM*, pages 747–758. SIAM / Omnipress, 2011.

[2] J. Zico Kolter and Matthew J. Johnson. REDD: a public data set for energy disaggregation research. In *SustKDD Workshop on Data Mining Applications in Sustainability*, 2011.

[3] J Zico Kolter and Tommi Jaakkola. Approximate inference in additive factorial HMMs with application to energy disaggregation. In *International Conference on Artificial Intelligence and Statistics*, pages 1472–1482, 2012.

List of Figures

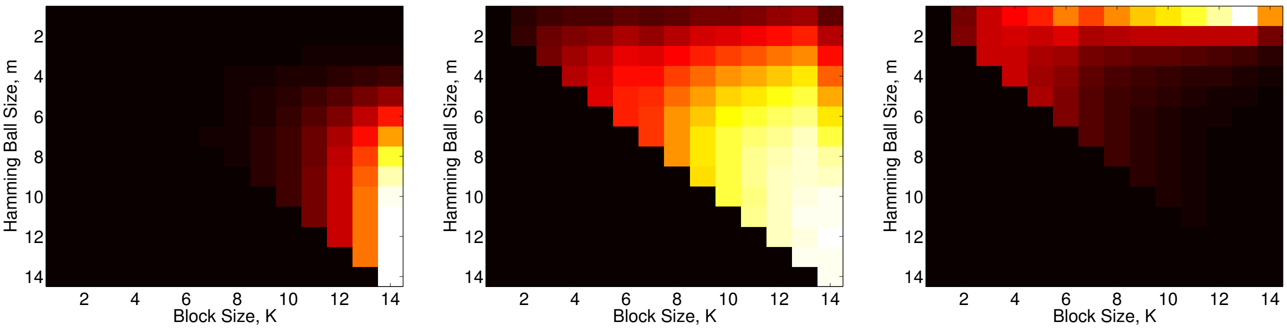

Complexity Sampling Efficiency Overall Efficiency

Figure 1: Sampling from a posterior distribution over $D = 14$ binary variables having two symmetric modes so that jumping between the modes requires simultaneously flipping two certain binary variables. The left panel shows the computational complexity for all block Hamming Ball schemes. The middle panel shows the corresponding sampling efficiency which was defined as the transition probability between the two modes (an ideal exact sampler has maximum transition probability equal to 0.5). Finally, the right panel displays the ratio between sampling efficiency and computational complexity which is maximized for $(m, K) = (1, 13)$. For all panels brighter colours indicate higher values.

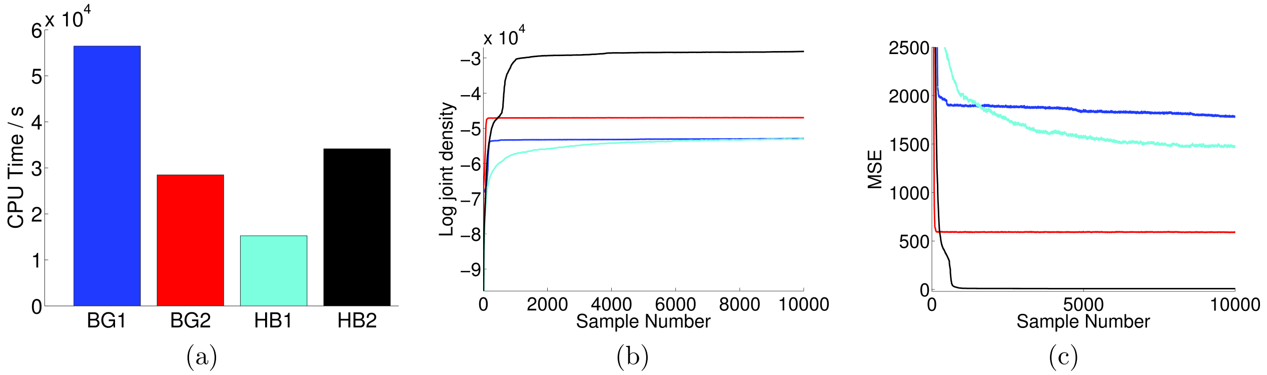

Figure 2: Performance in the training phase for the REDD dataset. Panel (a) shows the actual CPU times for carrying out 10,000 sampling iterations for fitting the FHMM to a sequence of length $9,600$ electricity measurements. Panel (b) shows the evolution of the log joint density while panel (c) gives the evolution of the MSE scores.

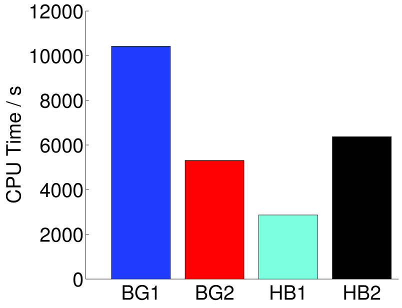

(a)

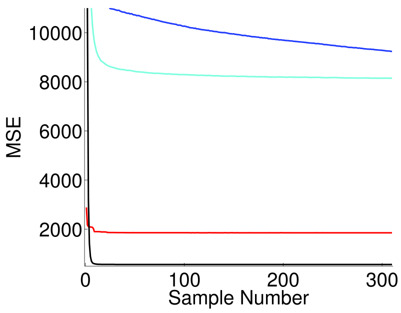

(b)

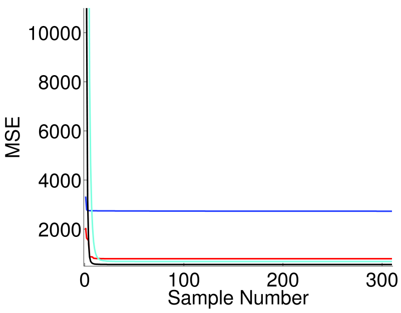

(c)

Figure 3: Performance of the prediction phase for the REDD dataset when each algorithm uses its own most representative sample over $(\mathbf{W}, \sigma^2)$ taken to be the last sample after 10,000 iterations in the training phase (panel b) and by using a common representative sample for all algorithms taken to be the last sample from HB2 (panel c). The panel in (a) shows the actual CPU times for carrying out 300 sampling iterations for analyzing the full test sequence of length 57,600.

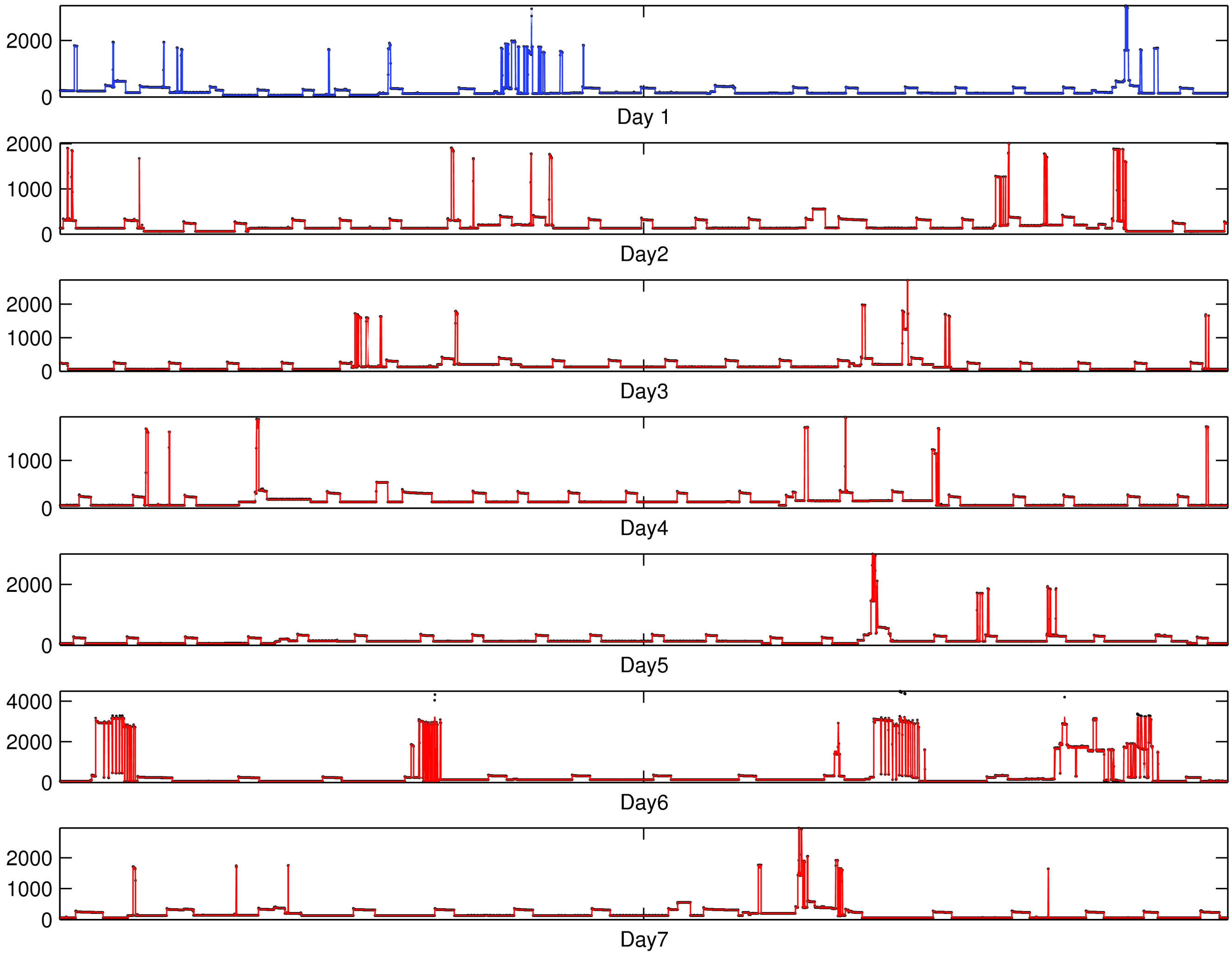

Figure 4: Performance on the 7 day REDD data set. Each row displays the actual values of the electricity measurements in the REDD dataset (black dots) together with the mean prediction/reconstruction found by the HB2 algorithm (blue or red). Specifically, the panel in the first row shows the reconstruction in the training sequence, corresponding to Day 1, that was considered for training the FHMM, while the remaining 6 panels show the reconstructions of the full test sequence broken down to individual days.
